# Fibroblast growth factor receptor signaling as therapeutic targets in female reproductive system cancers

**DOI:** 10.7150/jca.44727

**Published:** 2020-10-21

**Authors:** Dong-Li Zhu, Xiao-Mei Tuo, Yu Rong, Kun Zhang, Yan Guo

**Affiliations:** 1 Key Laboratory of Biomedical Information Engineering of Ministry of Education, Department of Trauma Surgery, Honghui Hospital, College of Medicine, Xi'an Jiaotong University, Xi'an, Shaanxi, P. R. China.; 2 Biomedical Informatics & Genomics Center, School of Life Science and Technology, Xi'an Jiaotong University, Xi'an, Shaanxi, P. R. China, 710054.; 3 Research institute of Xi'an Jiaotong University, Hangzhou, Zhejiang, P. R. China, 311215.

**Keywords:** Fibroblast growth factor, Fibroblast growth factor receptor, Genetic variations, Therapeutic targets, Female reproductive system cancer

## Abstract

Ovarian cancer, cervical cancer and endometrial cancer are three relatively common malignant cancers of the female reproductive system. Despite improvements in female genital tract cancer detection and development of new therapeutic approaches, there are still poor prognoses and some do not respond to therapeutic patterns, displaying low survival and high frequency of recurrence. In an era of personalized medicine, novel therapeutic approaches with greater efficacy for these cancers represent an unmet need. One of the actionable signaling pathways is the fibroblast growth factor receptor (FGFR) signaling pathway. Several mutations and alterations in FGF/FGFR family members have been reported in human cancers. FGF/FGFR signaling pathway has become a new target for cancer therapy. This review will summarize the role of FGFR pathway and the genetic alterations of the FGF/FGFR related to female reproductive system cancer. We will describe the available inhibitors of FGFR pathway for potential treatment of female reproductive system cancer. Furthermore, we will discuss FGFR-targeted therapies under clinical development for treatment of female reproductive system cancer.

## Introduction

Endometrial cancer, ovarian cancer and cervical cancers are three relatively common malignant tumors of the female reproductive system. Endometrial cancer that accounts for more than 95% of cases of uterine cancer is one of the most prevalent forms of gynecological cancers. It is thought to be caused by increasing estrogen levels relative to progesterone in the body. Endometrial cancer at stages I and II responds well to surgical interventions, but the disease at stages III and IV has poor prognosis with low survival rates. Ovarian cancer is the third leading gynecological malignancy worldwide and carries the highest mortality. Most ovarian cancers initiated from epithelial cells and are thus composed of poorly differentiated epithelial cells 1. Cervical cancer has the fourth greatest global burden of cancer among women for both incidence and mortality 2. WHO estimated that 570,000 new cases occurred and 311,000 women died from cervical cancer globally in 2018, with nearly 90% of these deaths occurring in low-income and middle-income countries 3. Cervical cancer is caused by infection with high-risk genotypes of human papillomavirus (HPV). Despite improvements in female genital tract cancer detection and development of new therapeutic approaches, there are still poor prognosis and non-response to therapeutic patterns, displaying low survival and high frequency of recurrence. Therefore, it is especially necessary for early diagnosis, treatment and survival of cancer patients to find specific biomarkers and drug targets for female reproductive system cancer.

Fibroblast growth factor receptors (FGFRs) are a family of receptor tyrosine kinase (RTKs) encoded by four different genes (FGFR1-4), among them, FGFR1-FGFR3 generate two major splice variants of immunoglobulin-like domain III, referred to as IIIb and IIIc, which are essential determinants of ligand-binding specificity 4. The signaling of FGFR axes is involved in cell proliferation, differentiation, tissue modeling, and angiogenesis via gene amplification, overexpression, point mutations or chromosomal translocations, which can lead to the development and/or progression of cancer 5. In the last few years, numerous studies have uncovered increasing evidence that FGFRs are driving oncogenes in certain cancers and act in a cell autonomous fashion to maintain the malignant properties of tumor cells. These observations make FGFRs increasingly attractive as targets for therapeutic intervention in cancer. Since FGFR inhibition can reduce proliferation and induce cell death in a variety of *in vitro* and *in vivo* tumor models harboring FGFR aberrations, an increasing number of researchers have selected FGFRs as targets for anticancer drug development 5.

Some of the most striking clinical findings regarding FGFRs relate to how these receptors are implicated in female genital tract cancers. In this article, we describe recent advances of FGFR signaling pathway in endometrial, ovarian and cervical carcinogenesis and progression. Moreover, we highlight the genetic variations (including somatic mutation and gene amplification) of FGF or FGFR family members and summarize the FGFR-targeted therapies under clinical development for treatment female genital tract cancers.

## FGF/FGFR signaling pathway

FGFRs in response to fibroblast growth factors (FGFs) transmit signals. The FGF family belongs to a large family of growth factors with significant expression profiles in the female reproductive tract and potentially important roles on fertility. This family is composed of 18 secreted proteins that are grouped into 5 subfamilies according to sequence homology 6,7. Both FGFs and FGFRs are involved in different physiologic processes, such as regulation of angiogenesis, embryonic development, and wound repair, among others. Additionally, the FGF/FGFR signaling network plays a critical role in cancer cell proliferation, survival, differentiation, migration, and apoptosis 8-10.

**Figure 1:**
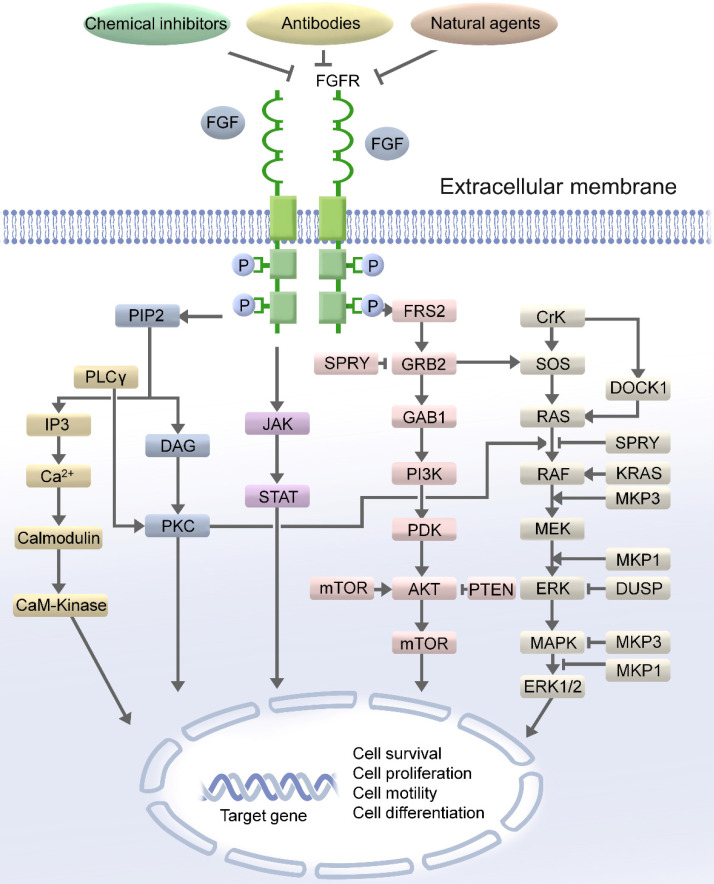
** The FGF/FGFR signaling pathway.** FGF/FGFR exhibits its physiological functions by regulating the main downstream signaling pathway, such as RAS/MAPK and PI3K/AKT/mTOR, FGF/FGFR signaling pathway could be blocked by the chemical inhibitors of FGF/FGFR, antibodies and natural agents. Therefore, targeting FGF/FGFR pathway could be an effective approach for the treatment of female reproductive system cancer patients.

Notably, dysregulation of the FGFR pathway is associated with various human cancers and is considered as an oncogenic signaling pathway 8, 11. In recent large-scale high-throughput studies, the dysregulation of FGFRs was detected in over 7% of cancers 12. FGFRs activated the propagation of signals through the receptor tyrosine kinase-mediated phosphorylation of the adaptor proteins of the pivotal cellular signaling pathways 13. The complex downstream signaling including the mitogen activated protein kinase (MAPK) 14, phospholipase Cγ (PLCγ) 15, signal transducer and activator of transcription (STAT), phosphoinositide 3-kinase (PI3K)/protein kinase B (AKT)/mammalian target of rapamycin (mTOR), and Ras-Raf-MEK-ERK pathway 16-19. FGFR substrate 2F (FRS2) is a key adaptor protein that associates with the receptor and initiates downstream signaling. The phosphorylation of FRS2 by FGFRs stimulates the binding of the growth factor receptor bound-2 (GRB2) protein to FRS2. GRB2 propagates signals through the two major signaling pathways: PI3K/AKT/mTOR and Ras-Raf-MEK-ERK (**Figure 1**).

### The cellular mechanisms of FGFR signaling

FGF/FGFR signaling governs fundamental cellular processes such as cell survival, proliferation, migration, differentiation, embryonic development, organogenesis, tissue repair/regeneration, and metabolism 10, 20, 21. Notably, FGFR2 expression has been reported to be associated with cell growth and progression of cervical dysplasia 22, 23. FGFRs recruit stromal cells, which are essential participants in the growth and motility of ovarian cancer cells.

### The molecular mechanisms of FGFR signaling

**Figure 2:**
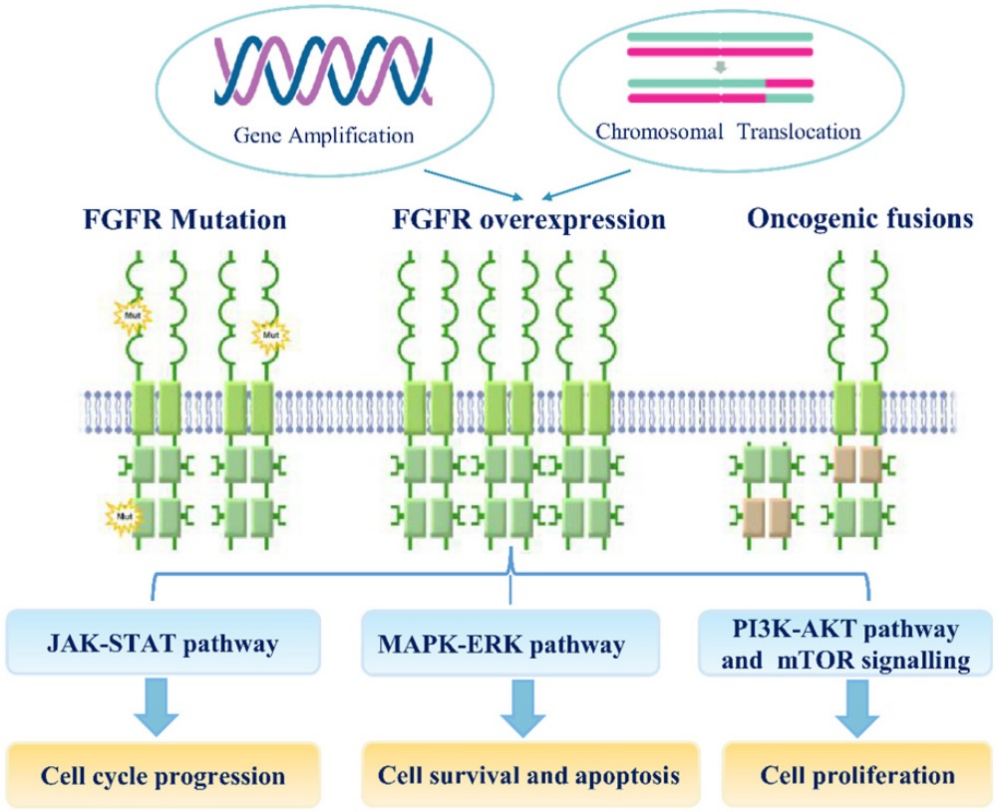
** The mechanisms of FGF/FGFR signaling dysregulation.** Enhanced FGFR signaling dysregulation is mediated by genetic alterations (Gene amplification, FGFR mutations, chromosomal translocations and gene fusions).FGFR over-expression by gene amplification or chromosomal translocation. FGF/FGFR signaling dysregulation can activate JAK/STAT, MAPK/ERK, PI3K-AKT and mTOR signaling pathways; these signaling can regulate multiple cellular processes, including cell survival, proliferation, motility apoptosis and so on.

Genetic variations, especially SNPs, and genomic alterations, such as gene amplification, chromosomal translocation, and point mutation, are involved in the transcriptional upregulation of FGFR mRNAs and the functional activation of FGFR proteins during carcinogenesis (**Figure 2**). Recent study suggested that the fibroblast FGF/FGFR family could interact with PI3K/AKT pathway and subsequently involve in the carcinogenesis of ovarian cancer. In endometrial cancer cell lines, loss of PTEN has been suggested as apotential mechanism of resistance to FGFR inhibition 71. Noticeably, co-treatment of FGFR2 and mTOR has a synergistic effect on the growth of endometrial cancer cell lines bearing an activating FGFR2 mutation, irrespective of PTEN status.

### FGFR signaling as a hot target for the development of anti-tumor drugs in clinical

**Figure 3:**
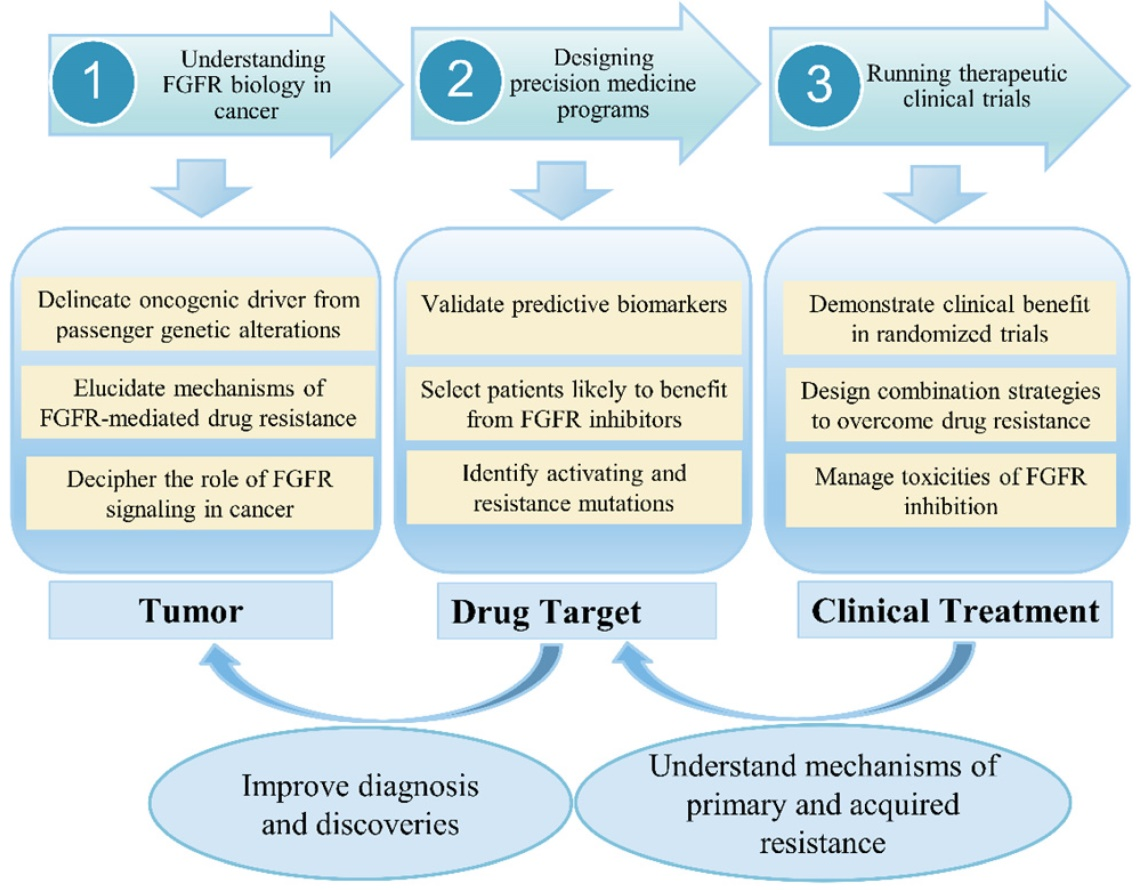
** FGFR signaling research from bench to bedside in cancer.** The challenges and prospects for the development of FGFR-targeted therapies.

In clinical, although anti-FGFR therapy represents a promising targeted cancer treatment, early phase clinical trials have had mixed success, with response to therapy dependent on several factors, including cancer type, tumor histology, and presence or absence of certain biomarkers 24. An increasing number of drugs against the FGF pathways is currently in clinical testing. Previous study revealed that combined inhibition of mTOR and FGFR signaling could be a promising anticancer approach 25. Choi *et al*. 26 investigated the clinical significance of FGFR1, FGFR2, FGFR3 and FGFR4 expression in a well-defined cohort of cervical cancers. One important conclusion is that a high expression of FGFR2, FGFR3, and FGFR4 showed longer disease-free survival and overall survival. In recent years, a large number of studies have confirmed that blocking FGF-FGFR-mediated tumor signaling pathway can effectively inhibit tumor proliferation and metastasis. Therefore, FGFR has become a hot target for the development of anti-tumor drugs. FGFR tyrosine kinase inhibitors (TKIs) have been undergoing Phase I/II clinical trials, but no inhibitors specifically targeting FGFRs have been marketed or used clinically. Since most of the kinase domains are highly similar, the first generation of FGFR TKIs (non-selective FGFR mainly including dovitinib, lucitanib, lenvatinib, ponatinib and nintedanib) generally inhibit various kinases other than FGFR (such as VEGFRs, PDGFRs, etc.). These non-selective inhibitors usually cause large toxic side effects and are greatly limited in practical applications. Second-generation FGFR inhibitors such as AZD4547, JNJ-42756493, BGJ-398 and LY2874455 have higher FGFR selectivity than first-generation inhibitors and are expected to produce higher levels of clinical benefit and reduce the risk of adverse reactions. At present, it has been found that selective inhibitors of FGFR4 (BLU554, H3B-6527 and FGF401) can effectively treat liver cancer, and can avoid some side effects caused by multi-target inhibitors. However, these drugs are still in the clinical research stage and have not been approved for clinical use by the FDA. In the context of low-frequency molecular aberrations, Prospective selection of patients with specific FGFR aberrations is one of the major challenges in clinical trials. However, molecular screening is therefore a crucial challenge for the development of FGFR inhibitors as patient selection is a key in this context (**Figure 3**). The main challenges have included (i) determining optimal diagnostic procedures for FGFR molecular alterations, and standardizing the definition of FGFRs amplification; (ii) detecting rare-frequency fusion genes involving various partners; (iii) discriminating between passenger and driver alterations; (iv) integrating the available information within a specific cellular and tumor heterogeneity context.

## FGFR signaling in female reproductive system cancer

### Cervical cancer

Cervical cancer is the fourth most common female malignancy worldwide. Each year, more than half a million women are diagnosed with cervical cancer and the disease results in over 300,000 deaths worldwide 27. Compared with the most affluent counties, mortality rates in the poorest counties were 2-fold higher for cervical cancer 28. The most common risk factor for the development of cervical cancer is chronic infection by oncogenic human papilloma virus (HPV), such as HPV16 and HPV18 viruses 29. Recently years, cervical cancer continues to be the second leading cause of cancer death in women aged 20 to 39 years, which underscores the need for increased HPV vaccination uptake in adolescents and guideline-adherent screening in young women 28.

The oncogenic significance role of the FGFRs has been elucidated in cervical cancer. Choi *et al*. 26 investigated the immunohistochemical expression of FGFR1, FGFR2, FGFR3, and FGFR4 in 336 cervical cancer patients, and confirmed that FGFR2, FGFR3, and FGFR4 expressions were important prognostic indicators in cervical cancer. Recent studies have shown the possible involvement of aberrant FGFR signaling with HPV16 E5 expression 30. FGFR2 expression has been reported to be associated with cell growth and progression of cervical dysplasia. The FGFR3-TACC3 fusion gene has been detected in uterine cervical cancer 22, 23. Recent study has identified four samples that harbored FGFR3-TACC3 fusion as an attractive therapeutic target through analyzing RNA sequencing data from 306 cervical cancer samples 31. However, FGFR2 and FGFR3 mutations are rarely detected in cervical cancer 32, 33.

### Ovarian cancer

Ovarian cancer is the leading cause of death from gynecologic cancers 34. Advanced ovarian cancer remains an unmet clinical need. Compared with current treatments, potential targeted therapies could be more effective. Recent study suggested that the FGF/FGFR pathway can interact with PI3k/AKT pathway involved in the carcinogenesis of ovarian cancer 35. Individual FGFRs including FGFR2 IIIb are over-expressed in ovarian cancer 36. Taniguchi F *et al*. 37 found that up-regulated FGFR2 expression has been shown to be potentially involved in the transformation of ovarian endometrioma to clear cell carcinoma of the ovary. Although FGFR2 mutations are considered rare in ovarian cancer, the Ser252Trp mutation seen in endometrial cancer has also been detected in the ovary cancer 38, suggesting a loss of ligand specificity for FGF signaling in at least some ovarian cancers.

FGFR4 is a prognostic marker for advanced-stage, high-grade serous ovarian cancer. Experiments *in vitro* and *in vivo* have shown that silencing FGFR4 and inhibiting ligand-receptor binding significantly decreased the proliferation, survival, and invasiveness and increased apoptosis of ovarian cancer cells, suggesting that FGFR4 protein expression is a new therapeutic modality for this disease and will improve its survival in clinical trials 39. High levels of FGFR4 protein have been reported in serous ovarian carcinomas and were associated with poor patient survival 39, although the ligand involved was suggested to be FGF19 40. On the other hand, a Gly388Arg mutation in FGFR4 has been reported in ovarian cancer and was associated with increased patient survival 41.

### Endometrial cancer

Endometrial cancer is the most common gynecologic malignancy among women in developed countries, with an estimated 63,230 new cases in 2018 42. Most patients are peri-and post-menopausal. In spite of the relative survival of 5 years is high for patients diagnosed with early-stage endometrial cancer, however, there are few treatment methods for patients who present with metastatic or recurrent endometrial cancer, and the prognosis of such patients remains poor. Recently, molecular targeted therapies have shown promising results in the management of endometrial cancer. FGFR pathway is one of pathways related to the pathogenesis and progress of endometrial cancer 43. FGFR alterations reported in endometrial cancers most frequently involve FGFR2 12. According to The Cancer Genome Atlas (TCGA) data reported in Nature in 2013, the most frequently altered FGFR gene in endometrial cancers was FGFR2, with 12.5% of sequenced samples (n=248 samples) harboring alterations in this gene. Recently, several independent studies have identified FGFR2 mutations in endometrial cancer 44-46. FGFR2 is thought to be a potential therapeutic molecular target for patients with FGFR2 activation-associated endometrial cancer. In endometrial cancer cell lines harboring activating FGFR2 mutations, inhibition of FGFR kinase activity inhibited cell cycle progression, cell survival, and colony formation. *In vivo* experiment has shown that FGFR inhibitor decreased the growth of FGFR2-mutated endometrial cancer xenograft models 44, 47. As the FGFR small-molecule inhibitor PD173074 diminishes survival and anchorage-independent growth by endometrial cancer cell lines expressing activating FGFR2 mutations 44. Previous studies have revealed that combined inhibition of mTOR and FGFR signaling could be a promising anticancer approach in endometrial cancer 25.

## The genetic variations and somatic mutations of FGF/FGFR pathway

The FGF/FGFR signaling pathway is frequently deregulated in human cancers. Over the last years, several mutations and alterations in FGF-FGFR pathway have been reported in cancer 48. Helsten* et al*. 12 sequenced 4853 tumor tissue samples, and found that 7.1% of all samples had genetic alterations in the FGF-FGFR axis. We summarized the germline genetic alterations (amplification and Single-nucleotide polymorphisms) as well as somatic changes (somatic mutation and somatic copy number alterations) of FGF and FGFR family members in **Table 1.**

### FGFR genetic alteration in female reproductive system cancer

Previous studies of germline FGFR mutations indicate that point mutations can result in differential localization and signaling 49-53. Single Nucleotide Polymorphisms (SNPs) of FGF or FGFR are associated with increased risk of female reproductive system cancer 54-57. Somatic mutations in FGFR1, FGFR2, FGFR3, and FGFR4 genes are reported in endometrial cancer. FGFR1 and FGFR3 amplifications are also found in endometrial cancer through integrating genomic characterization of somatic copy number alterations 58. FGFR2 mutations are the most frequent genomic aberration and have been found in 10-12% of endometrial carcinomas 12, 44. Activating FGFR2 mutation could be an important therapeutic target for endometrial cancers. Decreased cancer-specific survival is also seen in a multi-institutional cohort of over 950 endometrial cancer patients with somatic FGFR2 mutations, irrespective of stage 59. Furthermore, the copy number of FGFR1 was elevated both in endometrioid carcinoma and ovarian cancer 60. Mutations in FGFR3 are found in around 5% of cervical cancers 61, 62. Gene amplification in FGFR1, point mutation in FGFR2, and genetic alterations in FGFR3 are relatively frequent in ovarian and uterine cancer 12. Somatic mutations of the FGFR3 gene encompass the extracellular and transmembrane domains of the protein 63. To investigate oncogenic genetic alterations in cervical cancer, Xiang *et al*. 64 examined the mutational status of 16 oncogenic genes, such as FGFR2 and FGFR3 as well as FGFR3-TACC3, FGFR1-TACC1 fusions in a cohort of 285 Chinese patients with resected cervical cancer. Li *et al*. 56 found that FGFR4 gene polymorphism rs351855 (Glu388Arg) was associated with the prognosis of high-risk HPV infection cervical cancer, and the risk of worse prognosis inpatients with allele A was higher than that of GG patients.

### FGF genetic variation in female reproductive system cancer

Besides FGFR aberrations, a number of studies have also shown that genetic variations of FGF are associated with the disease. Take FGF2 for example, our group previously found that the polymorphisms of FGF2 gene are significantly associated with obesity and osteoporosis in Chinese population 65, 66. Meng *et al*. 55 investigated the genetic variants in FGF-FGFR pathway for associations with risk of ovarian cancer, therapeutic and overall survival. They revealed FGF2 rs167428 as the primary factor contributing to overall survival. FGF23 rs7961824 showed the most significant association with ovarian cancer survival. This provides a molecular approach for monitoring therapeutic response, and prediction of cancer diagnosis.

## FGFRs as therapeutic targets

Since FGF/FGFR signaling plays a crucial role in cancers, a variety of small molecule FGFR inhibitors target FGF/FGFR signaling pathway have been developed and shown significant therapeutic effects in pre-clinical and clinical studies 14. FGFR inhibitors function as tumor suppressors, therefore, more efforts have been focused on developing the inhibitors to target FGFRs, which show particular promise as an anticancer monotherapy or an adjunct treatment. There are mainly two kinds of FGFR inhibitors in clinical trials, including non-selective FGFR TK inhibitors (TKIs) and selective FGFR TKIs. Another two anti-FGF/FGFR agents are neutralizing monoclonal antibodies (mAbs) and FGF ligand traps.

### Non-selective FGFR TKIs

Actually, the most clinically advanced compounds are non-selective TKIs, such as brivanib, dovitinib, lenvatinib, ponatinib, nintedanib, and cediranib. Most of these inhibitors target ATP binding pocket in the TK domains of FGFRs through reversible or covalent bonds 67. For example, ponatinib can impede the autophosphorylation activity of FGFRs by binding to the hinge region of FGFRs and block the ATP-binding cassette motif 68. Several TKIs which target the FGFR pathway have shown preclinical activity 25, 47, 69.

To date, the most clinically advanced FGFR TKIs in FGFR targeted treatment is dovitinib (TKI258), which is now being tested in endometrial cancer patients with FGFR2 mutation (NCT01379534). Brivanibis another dual TKI against FGFRs and VEGFRs, which was found to be effective in metastatic solid malignancies resistant to standard therapy and is currently being developed as an anti-angiogenic agent in Phase II clinical trials 70. Lenvatinib (E7080) shows inhibitory activity against VEGFRs, FGFRs and PDGFRs, which is being tested in Phase II clinical trials, examining the efficacy in patients with metastatic endometrial cancer (NCT01111461) 71, 72. Nintedanib (BIBF 1120) is an orally administered triple angio-kinase inhibitor of the receptors of VEGFR-1-3, PDGFR-a/b and FGFR-1-3 73.

AL3818 (anlotinib) is a receptor tyrosine kinase inhibitor targeting vascular endothelial growth factor receptors (VEGFR1, VEGFR2/KDR, and VEGFR3), stem cell factor receptor (C-kit), platelet-derived growth factor (PDGFA), and fibroblast growth factor receptors (FGFR1, FGFR2, and FGFR3). This study evaluates the efficacy of AL3818 studying tumor regression in endometrial cancer model 74.

Several other TKIs are also summarized in Table 2, including cediranib, ponatinib, ENMD2076, pazopaniband regorafenib, which have been developed and are in pre-clinical and clinical evaluation 75. However, almost all of these non-selective compounds induce a series of toxic effects, such as cardiotoxicity or proteinuria due to the concurrent VEGFR inhibition, cutaneous reactions, digestive disorders, and gastrointestinal diseases 24. As far as we know, there is no information about its activity against FGFR, therefore, a new clinical trial has been initiated with the aim of more substantial proof (**Table 2**).

### Selective FGFRs TKIs

AZD4547 is a famous selective TKI that specific target for FGFRs (FGFR1-3) 76. The inhibitory activity of AZD4547 has been demonstrated *in vitro* and *in vivo* models of endometrial cancer characterized by FGFR activation due to genetic alteration 77. BGJ398 is also a selective reversible ATP-competitive FGFR1-3 inhibitor. Its anti-tumor activity was firstly assessed in pre-clinical models of endometrial cancer 47, 78. The FGFR inhibitors of BGJ398 and AZD4547are mostly in early-phase development programs (**Table 3**). Phase II trials with genomic enrichment are ongoing. A number of clinical trials use AZD4547 in cancer. One recent finding revealed that combined treatment of BGJ398 and rapamycin may be a promising therapeutic strategy in the treatment of patients with ovarian cancer 25. They also investigated whether the combined inhibition of mTOR and FGFR pathways would enhance the anticancer effects in the treatment of ovarian cancer. JNJ-42756493 (Erdafitinib) is a highly selective TKIs and shows inhibitory activity against FGFR1-4. Erdafitinib binds the ATP-pocket of the FGFR1 kinase domain with unique structural conformations and its inhibitory efficacy is reduced in the presence of the acquired gatekeeper mutation FGFR3 79. JNJ-42756493 suppresses FGFR phosphorylation and downstream signaling 80 and is able to induce stabilization of the disease in advanced solid tumors characterized by FGFR translocations or fusions (such as FGFR3-TACC3).

### Monoclonal antibodies and FGF ligand traps

Anti-FGFR mAbs as well as small molecules acting as traps for the ligands of the FGFR family might represent a new strategy for the treatment of tumors. Based on the identification and characterization of FGF ligands, FGF ligand traps have allowed the development of promising FGF-targeting molecules with potential implications for the therapy of FGF-driven tumors 81. Since these drugs have higher specificity that may result in a reduced toxicity due to the absence of off-target effects. Whereas, there is only one anti-FGFR mAb (PRO-001) entered clinical trials in female reproductive system cancer. In addition, FP-1039 is a FGF ligand trap and currently in phase II clinical trials to treat endometrial cancer (**Table 3**). Pre-clinical cancer models with genetic aberrations in the FGFR pathway, including FGFR2-mutated endometrial cancer, are particularly sensitive to FP-1039 mediated tumor inhibition 82.

**Figure 4:**
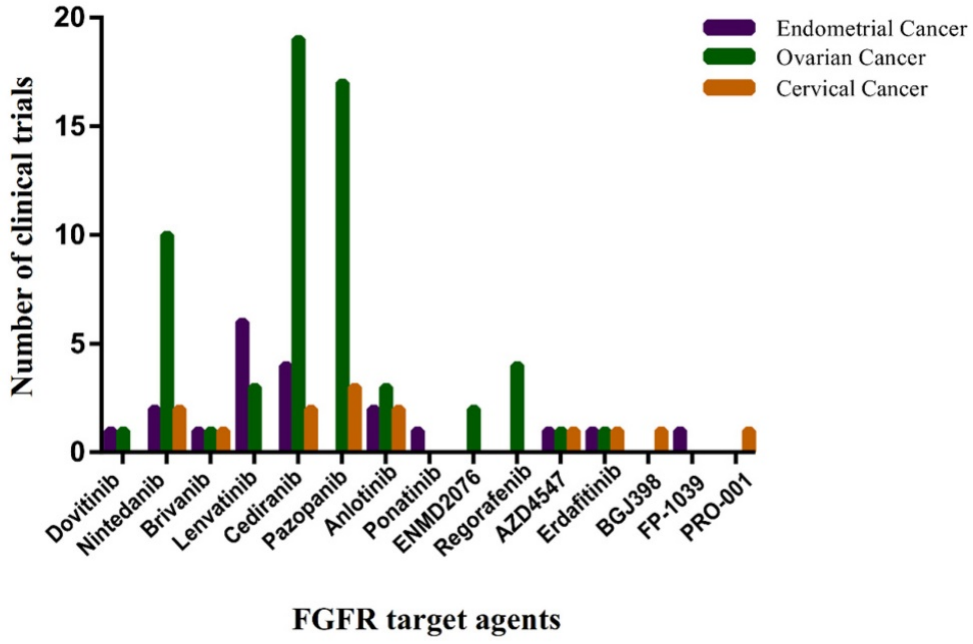
** The number of clinical trials of FGFR target agents in endometrial cancer, ovarian cancer and cervical cancer.** The horizontal axis represents Non-selective TK inhibitors, selective TK inhibitors, mAbs and FGF traps. The vertical axis represents the number of clinical trials. Date sourced from www.clinicaltrails.gov.

A large effort to develop FGF/FGFR inhibitors as anticancer treatments is underway. The most clinically advanced anti-FGFR drugs are small-molecule TKIs, some of them are monoclonal anti-FGFR antibodies and FGF-trapping molecules. Those anti-FGFR drugs that have entered the clinical phases of development are summarized in **Figure 4.**

## Conclusion

The cancers of the female genital tract represent a leading cause of morbidity and mortality among women worldwide. Undoubtedly, in the past decade, FGF/FGFR signaling therapies are under development for the treatment of gynecologic malignancies as well as in many other solid tumors. Therefore, dissecting canonical FGF/FGFR signaling pathways is still valuable. Deregulation of the FGF/FGFR signaling axis is observed in a wide variety of human cancers 83, 84. Efforts are needed to recognize patients most likely to benefit from FGFR inhibitors, to validate clinically useful companion diagnostics, to implement combination strategies, to overcome chronic toxicities, and to determine the most clinically relevant compound for registration. Considering the specific FGFR molecular alterations, especially many somatic mutations of FGFRs in female reproductive system cancer type, the experimental determination of which mutations play a causative role in tumorigenesis is currently the rate-limiting step to fully understanding the clinical implications of genomic data.

In conclusion, targeting FGFR is a promising strategy in the treatment of female reproductive system cancer. It is plausible to hope that in the following years the research efforts in pre-clinical and clinical fields allow to establish an optimal treatment strategy in FGFR-addicted female reproductive system cancer population.

## Figures and Tables

**Table 1:** Genetic variations and somatic mutations of FGF and FGFR family genes in female reproductive system cancers

Gene	Cancer type	Germline or somatic	Genetic changes	Mutations SNPs	Refs
FGFR1	Ovarian cancer	Somatic	Gene amplification		85
FGFR1	Endometrial Cancer	Somatic	Gene amplification		86
FGFR2	Endometrial cancer	Somatic	Missense mutation	D101Y, S252W, P253R, C382R, N549K, N550K, L764fs*4	44, 45, 87-92
FGFR2	Cervical cancer	Somatic	Missense mutation	K660M	44, 93
FGFR2	Ovarian cancer	Germline	SNPs	rs4752566, rs2981582, rs3135830, rs2981451, rs3135826, rs2912759	55
FGFR3	Cervical cancer	Somatic	Missense mutation	S249C	61
FGFR3	Ovarian cancer	Somatic	Gene amplification		12
FGFR4	Cervical cancer	Germline	SNP	rs351855	56
FGF2	Ovarian cancer	Germline	SNPs	rs167428, rs308441	55
FGF2	Endometrial cancer	Germline	SNPs	754C/G	54
FGF23	Ovarian cancer	Germline	SNPs	rs7961824, rs12812339	55
FGF1	Ovarian cancer	Germline	SNPs	rs7727832, rs2052006, rs10070885. rs17099029	55

**Table 2:** ** Non-selective FGFR tyrosine kinase inhibitors of female reproductive system cancer**. Known targets, cancer type, clinical trials identifier FGFR criteria, phase and clinical trials involving multi-target TKIs with FGFR status bases patient selection are indicated for each compound. Data were collected at clinicaltrials.gov, cancer.gov and PubMed

Compound	Target	Cancer type	Clinical trial identifier	FGFR Criteria	Phase	Status
Dovitinib (TKI258)	FGFR3, VEGFR1/3, FGFR1, PDGFR3,	Endometrial Cancer	NCT01379534	FGFR2 mutation	II	Completed
		Ovarian Cancer	NCT01831726	FGFR 1-3 mutations or amplifications	II	Completed
Nintedanib (BIBF 1120)	VEGFR, FGFR, PDGFR	Endometrial Cancer	NCT02730416		II	Recruiting
			NCT01225887		II	Completed
		Ovarian Cancer	NCT01485874 NCT01329549		I	Terminated
			NCT01610869		II	Active, not recruiting
			NCT01583322 NCT00710762 NCT01669798		II	Completed
			NCT01314105 NCT02835833		I	Completed
			NCT01015118		III	Completed
			NCT02866370		II	Recruiting
		Cervical Cancer	NCT02009579		II	Recruiting
			NCT02835833		I	Completed
Brivanib (BMS-582664)	VGFR1-3, FGFR1-3	Endometrial Cancer	NCT00888173	FGFR2 activation mutation	II	Completed
		Ovarian Cancer	NCT03895788		I	Recruiting
		Cervical Cancer	NCT01267253		II	Completed
Ponatinib (AP24534)	VEGFR2-3, FGFR1-2	Endometrial Cancer	NCT01888562	FGFR2 mutation	Not Applicable	Withdrawn
Lenvatinib (E7080)	VEGFR2, FGFRs, PDGFRs	Endometrial Cancer	NCT01111461		II	Completed
			NCT03517449 NCT03884101		III	Recruiting
			NCT03005015		II	Withdrawn
			NCT02788708 NCT03006887		I	Active, not recruiting
		Ovarian Cancer	NCT01133756		I/II	Terminated
			NCT02788708		I	Active, not recruiting
			NCT03797326		II	Recruiting
Cediranib (AZD2171)	VEGFR1-3, FGFRs	Endometrial Cancer	NCT03660826 NCT03570437		II	Recruiting
			NCT01132820		II	Completed
			NCT01065662		I	Active, not recruiting
		Ovarian Cancer	NCT02889900 NCT03314740 NCT02345265		II	Active, not recruiting
			NCT02340611 NCT00278343 NCT00275028		II	Completed
			NCT00532194		III	Unknown
			NCT02681237		Not Applicable	Recruiting
			NCT03278717		III	Recruiting
			NCT03117933 NCT03699449		II	Recruiting
			NCT02855697		Early Phase 1	Recruiting
Cediranib (AZD2171)	VEGFR1-3, FGFRs	Ovarian Cancer	NCT01131234, NCT00475956		I	Completed
			NCT01065662		I	Active, not recruiting
			NCT01116648		I/II	Active, not recruiting
			NCT02446600		III	Active, not recruiting
			NCT02502266		II/III	Recruiting
			NCT02484404		I/II	Recruiting
		Cervical Cancer	NCT01065662		I	Active, not recruiting
			NCT01229930		II	Completed
ENMD2076	FGFR1-2, PDGFRs, VGFR2	Ovarian Cancer	NCT01104675		II	Completed
			NCT01914510		II	Completed
Pazopanib (GW786034)	VEGFR1-3, PDGFR, FGFR1/3	Ovarian Cancer	NCT01600573 NCT01402271		I/II	Unknown
			NCT01238770		I/II	Completed
			NCT02383251 NCT01610206		II	Active, not recruiting
			NCT01608009		I	Completed
			NCT01262014 NCT01644825 NCT00281632 NCT01468909 NCT01227928 NCT00561795		II	Completed
			NCT02055690 NCT01035658		I/II	Terminated
Pazopanib (GW786034)	VEGFR1-3, PDGFR, FGFR1/3	Ovarian Cancer	NCT00866697		III	Completed
			NCT01392352		II	Terminated
			NCT02009449		I	Active, not recruiting
		Cervical Cancer	NCT02348398		II	Withdrawn
			NCT00430781		II	Completed
			NCT01392352		II	Terminated
Regorafenib (BAY73-4506)	VEGFR1-3,PDGFRβ, FGFRs	Ovarian Cancer	NCT02278783		II	Terminated
			NCT02736305 NCT02584465 NCT02307500		II	Recruiting
Anlotinib (AL3818)	VEGFR1-3, PDGFR-α, FGFR1-3	Endometrial Cancer	NCT02558348	FGFR1, FGFR2 or 3 amplification or mutation	I/II	Recruiting
			NCT02584478		I/II	Recruiting
		Ovarian Cancer	NCT03924882		II	Recruiting
			NCT02558348	FGFR1, 2 or 3 amplification or mutation	I/II	Recruiting
			NCT02584478		I/II	Recruiting
		Cervical Cancer	NCT02558348	FGFR1, 2 or 3 amplification or mutation	I/II	Recruiting
			NCT02584478		I/II	Recruiting

Note: FGF, fibroblast growth factor; FGFR, fibroblast growth factor receptor; VGFR, vascular growth factor receptor; VEGFR, vascular endothelial growth factor; PDGFR, platelet-derived growth factor receptor.

**Table 3:** Selective FGFR TKIs, ligand trap and mAbs of female reproductive system cancer

Compound	Target	Cancer type	Clinical trial identifier	FGFR Criteria	Phase	Status
**Selective FGFR TKIs**						
AZD4547	FGFR1-3	Endometrial Cancer; Ovarian Cancer; Cervical Cancer	NCT02465060	FGFR1-3 mutation or translocation	III	Recruiting
BGJ398	FGFR1-3	Cervical Cancer	NCT02312804		I	Withdrawn
Erdafitinib (JNJ-42756493)	FGFR1-4	Endometrial Cancer; Ovarian Cancer; Cervical Cancer	NCT02465060	FGFR amplification/mutation or fusion	II	Recruiting
**FGF and traps and mAbs**						
FP-1039 (GSK3052230)	FGF2	Endometrial Cancer	NCT01244438	FGFR2 mutation	II	Withdrawn
PRO-001	FGFR3-specific blocking antibody	Cervical Cancer	NCT01263327		I	Completed

## Abbreviations

FGF: Fibroblast Growth Factor
FGFR: Fibroblast Growth Factor Receptor
PI3K: phosphoinositide-3-kinase
MAPK: mitogen-activated protein kinase
PKC: protein kinase C
PLCγ: phospholipase Cγ
HPV: human papilloma virus
TCGA: The Cancer Genome Atlas
FRS2: FGFR substrate 2F
GRB2: growth factor receptor bound-2
TKI: tyrosine kinase inhibitors
mAbs: Monoclonal antibodies
